# Effect of human platelet-rich fibrin lysate on collagen type I, collagen type III, and matrix metalloproteinase 1: a protocol study on rat models with pelvic organ prolapse

**DOI:** 10.12688/f1000research.152876.2

**Published:** 2025-09-16

**Authors:** Akbar Novan Dwi Saputra, Dicky Moch Rizal, Nandia Septiyorini, Muhammad Nurhadi Rahman, Yohanes Widodo Wirohadidjojo, Dwi cahyani Ratna Sari, Raden Mas Sonny Sasotya

**Affiliations:** 1 Department of Obstetrics and Gynecology, Faculty of Medicine, Public Health and Nursing, Dr. Sardjito Hospital, Universitas Gadjah Mada, Yogyakarta, Special Region of Yogyakarta, 55281, Indonesia; 2 Doctoral Program, Faculty of Medicine, Public Health and Nursing, Universitas Gadjah Mada, Yogyakarta, Special Region of Yogyakarta, 55281, Indonesia; 3 Department of Physiology, Faculty of Medicine, Public Health and Nursing, Universitas Gadjah Mada, Yogyakarta, Special Region of Yogyakarta, 55281, Indonesia; 4 Department of Dermatovenerology, Faculty of Medicine, Public Health and Nursing, Dr. Sardjito Hospital, Universitas Gadjah Mada, Yogyakarta, Special Region of Yogyakarta, 55281, Indonesia; 5 Department of Anatomy, Faculty of Medicine, Public Health and Nursing, Universitas Gadjah Mada, Yogyakarta, Special Region of Yogyakarta, 55281, Indonesia; 6 Urogynecology and Reconstruction Division, Obstetrics and Gynecology Department, Faculty of Medicine, Universitas Padjadjaran, Bandung, West Java, 45363, Indonesia

**Keywords:** Pelvic organ prolapse, human platelet-rich fibrin lysate, collagen type I, collagen type III, matrix metalloproteinase 1, extracellular matrix, protocol study

## Abstract

**Background:**

Pelvic organ prolapse (POP) is a prevalent condition caused by weakened pelvic floor support structures. Extracellular matrix alterations, including changes in collagen type I, collagen type III, and matrix metalloproteinase 1 (MMP-1), contribute to the pathogenesis of this condition. Human platelet-rich fibrin lysate (hPRF-L) is a novel regenerative treatment that has shown beneficial results in treating structural weaknesses related to various pelvic floor diseases, including POP.

**Methods:**

This study protocol aims to investigate the effects of hPRF-L injection on collagen I, III, and MMP-1 in the vaginal mucosa of a rat POP model. POP will be induced in female Sprague-Dawley rats, which will be randomly assigned to control, sham, and hPRF-L treatment groups. The hPRF-L group will receive weekly injections of hPRF-L (25, 50, or 75 μL) into the vaginal mucosa for 4 weeks. Vaginal tissue samples will be collected, and collagen type I, collagen type III, and MMP-1 expression will be evaluated using quantitative reverse transcription polymerase chain reaction and immunohistochemical analyses. Data analysis will be performed with ANOVA and post-hoc tests.

**Discussion:**

The findings from this study protocol are expected to provide valuable insights into the mechanisms by which hPRF-L impacts the structural integrity of the pelvic floor. By elucidating these mechanisms, this study aims to inform future POP treatment strategies. The anticipated results are an increase in collagen type I and III expression and a reduction in MMP-1 levels in the hPRF-L treatment group compared to the control and sham groups. These outcomes could support the use of hPRF-L as a regenerative therapy for managing POP, offering a potential alternative to more invasive surgical interventions.

**Conclusion:**

The expected results will contribute to the development of less invasive treatments for POP, improving patient outcomes and quality of life.

## Introduction

Pelvic organ prolapse (POP) is a common and impairing disorder that affects millions of women globally and significantly impairs their quality of life. It is characterized by weakened or damaged pelvic floor support structures, including the muscles, ligaments, and connective tissues. This condition contributes to the descent of pelvic organs, including the uterus, bladder, and rectum, into the vaginal canal. Advanced age, number of pregnancies, family history, genetic predisposition, and persistent exertion of pressure have been recognized as risk factors for POP. ^1^ Studies have reported that the prevalence of POP in Indonesia ranges from 26.4% to 81.25%, and the majority of cases do not receive prompt diagnosis. The most common method for management in the majority of cases is surgical intervention. ^2^^,^^3^ The chance of requiring pelvic floor reconstruction surgery over one’s lifetime is between 11% and 19%, which imposes an enormous financial burden on patients and the healthcare system. ^4^

Pelvic floor reconstruction surgery is a well-established treatment for POP. However, it is associated with several risks that must be carefully considered. Surgical complications can include damage to intestines, bladder, rectum or other organs, excessive bleeding due to damaging a vein or artery, and blood clots. ^5^ Moreover, even if the procedure is successfully performed, the rate of recurrence or failure is still high. The recurrence rate of POP after surgery was 37.7%. ^6^ The introduction of meshes or grafts was prompted by the unacceptably high failure rate of surgery. The use of non-absorbable synthetic mesh resulted in a significantly reduced recurrence rate compared to absorbable synthetic mesh and biological alternatives. Nevertheless, problems associated with mesh are common, including erosions, wound granulation, and dyspareunia. ^7^ Considering the significant risks and limitations associated with surgical interventions, alternative and less invasive therapies that can effectively address the underlying causes of POP and provide long-lasting relief for patients are urgently needed.

POP has a complex pathophysiology and is associated with extracellular matrix composition (ECM) changes and imbalanced connective tissue remodeling. The ECM is the basis for the formation of ligaments, fascia, and the vaginal wall. It represents a complex of high-molecular-weight proteins, including collagen, elastin, and proteoglycans. ^8^ The vaginal and pelvic floor ECM are mainly composed of collagen fibers, particularly collagen type I and type III. These collagen subtypes play a crucial role in the structural integrity and biomechanical properties of tissues. In POP, the ratio of collagen type I to collagen type III is often imbalanced, leading to decreased tensile strength and compromised tissue support. ^9^^,^^10^ Aside from collagen composition changes, the activity of matrix metalloproteinases (MMPs), a family of enzymes that are responsible for ECM remodeling, is also altered in POP. Specifically, prolapsed tissues exhibit an upregulation of MMP-1, also known as interstitial collagenase. MMP-1 is involved in collagen fiber degradation, contributing to the weakening of the pelvic floor support structures. ^11^ Previous studies indicate that alterations in the extracellular matrix (ECM) are a major contributing factor to POP. Collagen fibers are the main component of the extracellular matrix (ECM), and they play a crucial role in providing support to connective tissues. Therefore, the restructuring of collagen fibers in the vaginal area might benefit women with POP. ^12^

Rodents such as rats are the most widely used animal model for research into the development of spontaneous POP in women, as well as for testing new therapeutic modalities with the aim of improving outcomes of existing therapies. The histological similarity between human and rodent vaginal tissue is one of the main considerations for using this animal model. There are several other advantages related to the use of mouse models, including ease of handling, short lifespan, relatively low cost, and ease of obtaining animal samples. Rats also have a predictable estrous cycle and relatively short gestation period, which makes POP development faster, facilitating the research process. ^7^^,^^13^ Animal models allow interventions and manipulations that are not possible in human subjects, such as injury induction or invasive tissue sampling, thus helping to study the pathophysiology and development of POPs in depth and comprehensively test potential new therapies before clinical trials.

While murine models have been extensively utilized in POP research, we selected the rat model for several critical methodological advantages. First, the anatomical dimensions of rat pelvic structures (vaginal length: 15-20 mm, diameter: 4-6 mm) provide superior accessibility for precise hPRF-L injection compared to mice (vaginal length: 8-12 mm, diameter: 2-3 mm) [Reference needed]. Second, rats yield significantly larger tissue samples (approximately 3-4 fold greater mass), enabling comprehensive molecular analyses including RNA extraction, protein quantification, and histological evaluation from the same specimen. Third, the rat estrous cycle (4-5 days) more closely approximates human hormonal fluctuations compared to the mouse cycle (4-day cycle), providing better translational relevance for hormonal influences on extracellular matrix remodeling. Finally, previous pharmacokinetic studies have demonstrated that rat models show more predictable dose-response relationships for growth factor-based therapeutics, which is crucial for hPRF-L dosage optimization.

Although larger mammalian models demonstrate superior anatomical similarity to humans, rodent models remain the gold standard for initial mechanistic studies due to standardized protocols and ethical considerations. Specifically, rat vaginal tissue exhibits comparable collagen architecture to human tissue, with type I collagen comprising 70-80% of total collagen content and type III collagen representing 15-20%. The basement membrane composition and smooth muscle fiber orientation in rat vaginal tissue show similar patterns to human specimens, particularly in the distribution of elastin fibers and proteoglycan content. ^30^ However, we acknowledge that collagen cross-linking patterns and mechanical properties differ between species, which represents a limitation of this model that we will address in our discussion.

Platelet-rich fibrin (PRF) is a natural biomaterial generated from the patient’s own blood that is being used as a promising treatment for gynecological and pelvic floor disorders, including POP. It contains high concentrations of growth factors, cytokines, and other biologically active substances that have been proven to stimulate tissue regeneration, angiogenesis, and ECM remodeling. ^14^^,^^15^ Because of its ability to target the fundamental structural and functional defects associated with POP, the use of human platelet-rich fibrin lysate (hPRF-L) in treating POP has attracted considerable attention. The lysate form of PRF, which is obtained through platelet activation and lysis, contains a concentrated mixture of growth factors and bioactive molecules present in the original PRF material. ^16^

This protocol study aims to examine the effect of hPRF-L injection on collagen type I, collagen type III, and MMP-1 levels in the vaginal mucosa of a rat model with POP. In addition, this study provides useful insights into the therapeutic potential of hPRF-L for managing POP by exploring the mechanisms by which it may affect the composition and remodeling of vaginal tissues.

## Methods

### Study design

This prospective randomized controlled animal study will be designed to investigate the effects of hPRF-L injection on collagen type I, collagen type III, and MMP-1 expression in the vaginal mucosa of a rat model with POP. Collagen types I and III were selected as primary outcomes because they constitute >85% of vaginal wall collagen and directly correlate with tissue strength and elasticity. Type I collagen provides tensile strength, while type III collagen contributes to tissue flexibility and remodeling capacity. MMP1 was chosen as it specifically degrades fibrillar collagens (types I and III) and serves as a biomarker for ECM turnover. ^10, 11, 18^ The functional assessments will provide clinically relevant endpoints that translate directly to human applications.

**Figure 1.**
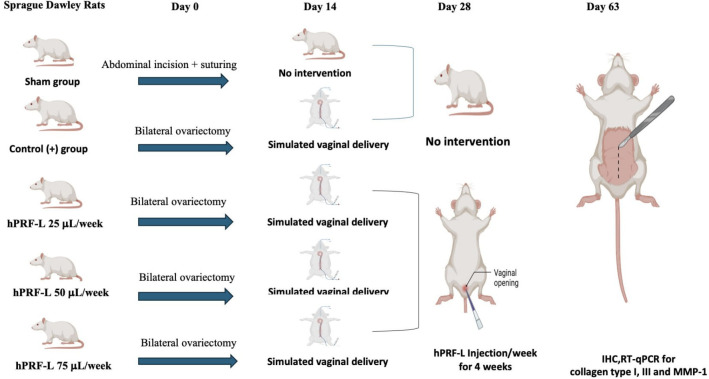
A visual illustration of the procedure for animal experiments.

The study will be conducted using female Sprague–Dawley rats, which are a well-established animal model for POP research. Thirty rats, aged 12–14 weeks and weighing between 200 g and 300 g, will be included. All animals will be nulliparous to eliminate confounding effects of pregnancy and delivery on baseline collagen metabolism and pelvic floor integrity. Nulliparous status will be confirmed by absence of mammary gland development and vaginal opening characteristics. This selection is based on evidence that pregnancy and vaginal delivery cause permanent alterations in collagen composition and MMP expression patterns. The animals will be kept in rooms that maintained a regulated temperature and humidity and followed a 12-h light/dark cycle. During the study, the rats will be provided unrestricted access to regular food and water. A sham group will be formed by randomly selecting six rats, and another six rats will be randomly selected as the control positive group. The remaining 18 rats will received an hPRF-L injection after POP induction. The hPRF-L treatment group will be further divided into three subgroups, with six rats in each subgroup receiving a different dosage of hPRF-L (25, 50, or 75 μL/week) for a duration of 4 weeks. Furthermore, a researcher who will be blinded to the specific treatment given to each rat will evaluate the outcomes. Figure 1 displays the study approach to the proposed research.

### Pelvic organ prolapse model in rats

Pelvic organ prolapse (POP) was established in all rats using a validated surgical procedure involving bilateral ovariectomy followed by simulated vaginal delivery, as described by Guo et al. This two-stage method combines hormonal depletion with mechanical stretch to induce tissue changes consistent with the pathophysiology of human POP. ^13^

It is important to note that due to the quadrupedal nature and small size of the rat model, it does not spontaneously present with anatomical prolapse or external “bulging symptoms” typically observed in human patients. Therefore, the success of the POP induction was not evaluated via an external grading scale. Instead, the model’s validity and the presence of POP were confirmed by a series of well-established, objective histopathological and biomechanical criteria consistent with the disease in humans. These criteria include significant alterations in tissue morphology, collagen content and composition (decreased Collagen I/III and increased I/III ratio), and a quantifiable increase in tissue stiffness (tangent modulus). The model also demonstrated elevated expression of matrix metalloproteinases (MMPs) and a corresponding decrease in their tissue inhibitors (TIMPs), which are key markers of the disease. This approach ensures that our study focuses on the fundamental underlying tissue pathology of POP and provides a robust, reproducible platform for evaluating the therapeutic effects of hPRF-L.

Stage 1: Bilateral Ovariectomy

1. Anesthesia: Rats will be anesthetized via intraperitoneal injection of ketamine (100 mg/kg body weight) and xylazine (10 mg/kg body weight). Depth of anesthesia will be confirmed by lack of pedal reflex.
2. Surgical preparation: The abdominal area will be shaved and disinfected with 70% ethanol followed by povidone-iodine solution. Sterile drapes will be used to maintain asepsis.
3. Ovariectomy procedure: a. A 2-cm midline incision will be made in the lower abdomen using sterile surgical scissors. b. The abdominal muscles will be separated along the linea alba. c. The ovaries will be identified and gently exteriorized. d. The fallopian tubes will be ligated with 4-0 absorbable sutures approximately 1 cm from each ovary. e. The ovaries will be excised using micro-surgical scissors. f. Hemostasis will be confirmed before closing the abdominal cavity. g. The abdominal muscles will be closed with 4-0 absorbable sutures in a continuous pattern. h. The skin will be closed with 4-0 non-absorbable sutures in an interrupted pattern.

Stage 2: Simulated Vaginal Delivery (2 weeks post-ovariectomy)

1. Anesthesia: As described in Stage 1.
2. Catheter preparation: a. A size 12 Foley catheter will be trimmed to remove the tip beyond the balloon. b. The catheter will be sterilized using alcohol.
3. Simulated delivery procedure: a. The sterilized catheter will be lubricated with sterile water-based gel. b. The catheter will be gently inserted into the vagina to a depth of approximately 1 cm. c. The balloon will be inflated with 2.5 mL of sterile 0.9% saline solution using a sterile syringe. d. The inflated catheter will be left in place for 4 hours. During this time, rats will be kept under light anesthesia. e. After 4 hours, the saline will be withdrawn from the balloon using a sterile syringe. f. The catheter will be gently removed.

### Preparation of human platelet-rich fibrin Lysate

The hPRF-L will be prepared from venous blood collected from one healthy female volunteer (age 25-35 years, nulliparous) following informed consent procedures approved by the Institutional Ethics Committee (Ref. No: KE/FK/0092/EC/2023). The following criteria will be used:

Inclusion criteria

• **Healthy female, aged 25-35 years:** To ensure a consistent and healthy donor pool, we will only include healthy female between 25 and 35 years old. This age range is selected to minimize variability associated with age-related physiological changes that could affect blood composition.
• **Nulliparous status:** We will only include nulliparous donor to eliminate the potential influence of pregnancy and childbirth on blood factors, which could alter the composition of the platelet-rich fibrin lysate (hPRF-L).
• **No history of pelvic floor disorders:** To avoid any confounding variables related to pre-existing pelvic conditions, we will exclude donor with a history of pelvic floor disorders.
• **No use of hormonal contraceptives or medications affecting coagulation:** The use of hormonal contraceptives or other medications that could impact blood coagulation will be an exclusion criterion to ensure the quality and consistency of the hPRF-L preparation.
• **Normal complete blood count and coagulation parameters:** Donor must have normal complete blood count and coagulation parameters to ensure the safety of blood donation and the quality of the hPRF-L product.

Exclusion criteria

• **Pregnancy or breastfeeding:** Pregnancy and breastfeeding are known to alter hormonal and physiological states, which could affect blood composition.
• **History of autoimmune disorders:** Autoimmune disorders can impact blood and inflammatory markers, making these donors unsuitable for providing a consistent hPRF-L product.
• **Anticoagulant therapy:** Donor undergoing anticoagulant therapy will be excluded to prevent any interference with the natural coagulation process required for hPRF-L preparation.
• **Active infection or inflammatory conditions:** Active infections or inflammatory conditions can alter the donor's blood composition, including cytokine and growth factor profiles, which could affect the therapeutic properties of hPRF-L.
• **Smoking or excessive alcohol consumption:** These lifestyle factors are known to influence overall health and blood parameters, and thus will be used as an exclusion criterion to maintain the consistency of the donor pool.

The hPRF-L used in this study will be prepared following a standardized protocol described by Choukron et al. ^17^ The process will begin with the collection of 20 mL of peripheral blood from the donor’s cubital vein. A trained phlebotomist will use a sterile 20 mL disposable syringe with a 21-gauge needle for blood collection. To prevent clotting and minimize platelet activation, the blood draw will be completed within 2 minutes. Immediately after collection, the blood will be transferred into sterile glass tubes (10 mL capacity, silica-coated) without the addition of anticoagulants. The tubes will be balanced and placed in a benchtop centrifuge (Centrifuge Hettich EBA 200) equipped with a fixed-angle rotor. Centrifugation will be performed at room temperature (20-25°C) for 10 minutes at 400 G.

This centrifugation process will result in the formation of two distinct layers within the tube: the upper layer comprising the PRF jelly and the lower layer containing the erythrocytes, indicating near-complete blood clotting after 5 minutes. The PRF jelly will be carefully removed from the tube using sterile tweezers. Next, sterile scissors will be used to cut at the border between the jelly and the erythrocytes. The isolated PRF jelly will then be transferred into a new sterile glass tube and incubated for 24 hours at 4 °C.

**Figure 2.**
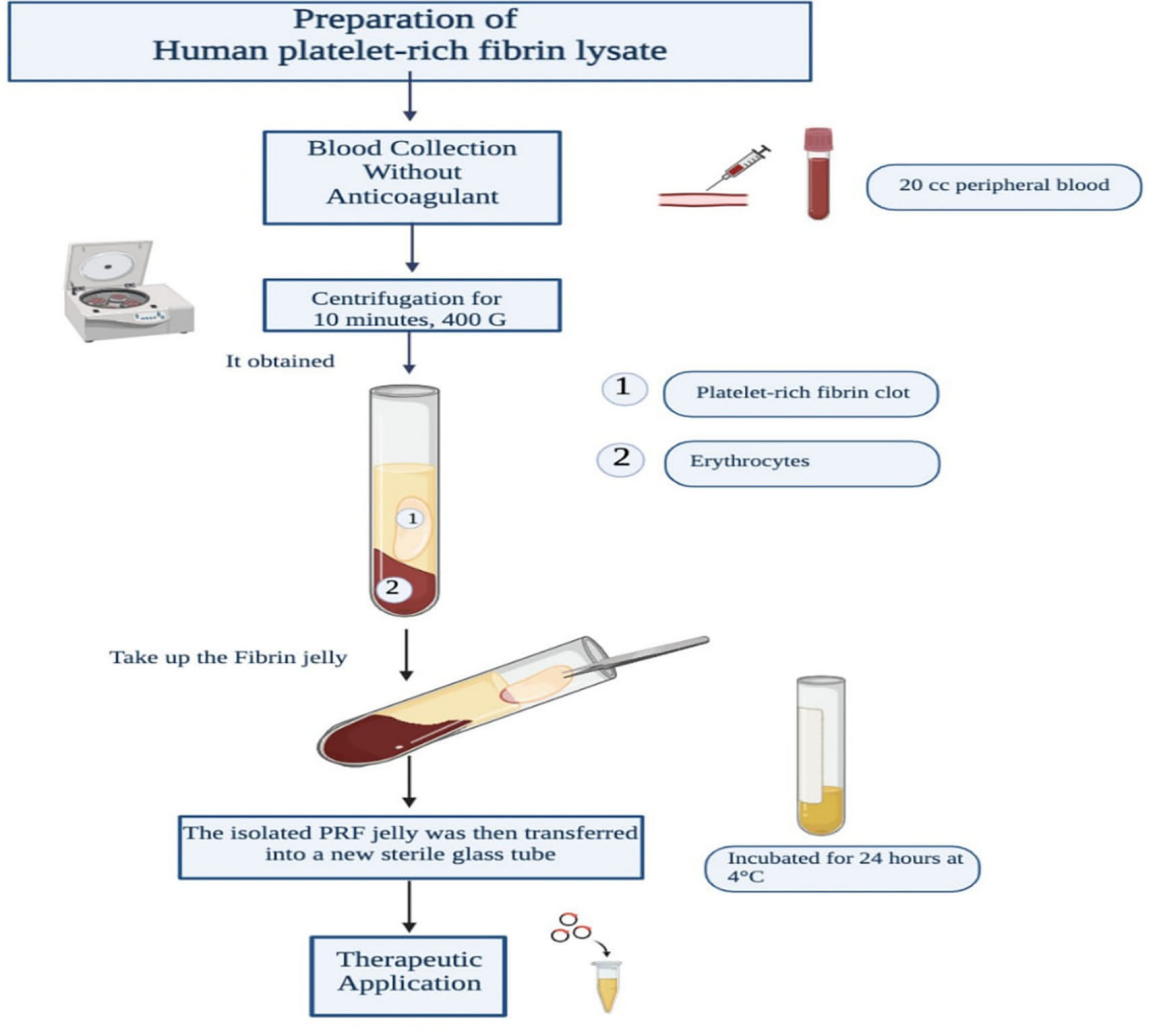
Preparation of human platelet-rich fibrin lysate.

Finally, the supernatant will be aspirated from the incubated PRF jelly and transferred into a 2 mL Eppendorf tube, where it will be stored at −20 °C until further use. The supernatant obtained through this process will constitute the PRF lysate that will be utilized in the present study. The preparation of human platelet-rich fibrin lysate is displayed in Figure 2.

Informed written consent will be obtained from all participants for the collection and use of their blood in this study. The consent process will be conducted as follows:

1. Potential participants will be provided with a detailed information sheet explaining the purpose of the study, the blood collection procedure, potential risks and benefits, and how their blood samples will be used.
2. A member of the research team will verbally explain the study and answer any questions the potential participants may have.
3. Participants will be given sufficient time (at least 24 hours) to consider their participation and ask any additional questions.
4. If they agree to participate, participants will be asked to sign a written consent form. This form will be available in both English and the local language to ensure full comprehension.
5. A copy of the signed consent form will be given to the participant, and another copy will be retained in the study records.

The use of written consent was approved by the Ethics Commission of FKKMK Gadjah Mada University (approval letter reference number: KE/FK/0092/EC/2023). Written consent was chosen over verbal consent to provide a clear record of the participants’ agreement and to ensure that all necessary information was provided consistently to each participant.

### Intravaginal human platelet-rich fibrin lysate injection procedure

Following the establishment of the POP model, the hPRF-L will be administered via intravaginal injection. To ensure accurate delivery of the full, precise dosage and to minimize animal distress, each rat will be anesthetized with a single intraperitoneal (IP) injection of ketamine at a dose of 0.1 mL per 100 grams of body weight. The injections will be performed by trained personnel to guarantee proper technique and complete dose administration at the specified vaginal sites. The use of anesthesia during this procedure is critical for ensuring the systematic and reproducible application of hPRF-L as outlined in the protocol.

After the rat model with POP is established, the researchers will administer hPRF-L injections into the vaginal mucosa layer. The injection protocol will involve delivering doses of 25, 50, or 75 μL of hPRF-L per week, for a duration of 4 weeks. To ensure even distribution, the injection sites will be rotated each week. The injection in the first week will be made in the 6 o’clock position, that in the second week will be made in the 12 o’clock position, that in the third week will be made in the 3 o’clock position, and that in the fourth week will be made in the 9 o’clock position.

hPRF-L will be injected directly into the subepithelial space of the vaginal wall using a 26-gauge syringe and maintaining a depth of 1–2 mm. The injection sites will be carefully selected at the midline of the anterior, posterior, right, and left lateral vaginal walls, approximately 0.2 cm from the vaginal opening. Moreover, the researchers will perform needle retraction and injection simultaneously to further facilitate the even distribution of the PRF lysate within the vaginal wall.

### Tissue harvesting and processing

The rats will be sacrificed 1 week after the last hPRF-L injection (series 4), and the vaginal wall will be removed. We will conduct the euthanasia through cervical dislocation after inducing deep anesthesia with a ketamine overdose. After euthanasia, the rat’s abdominal cavity will be opened, and the pubic symphysis will be disarticulated. First, the vaginal opening will be isolated from the surrounding perineal skin. Then, the vaginal tube will be isolated intact. The vaginal wall will be cut transversely into proximal and distal segments. The proximal segment will be immediately immersed in 10% neutral buffered formalin, processed, and embedded in paraffin blocks for histological evaluation. Meanwhile, the distal segment will be stored at −80 °C for molecular studies. The slides will be subsequently analyzed by two independent observers who will be blinded to the grouping and intervention.

At sacrifice (4 weeks post-treatment), vaginal tissues will be collected using standardized protocol:

1. **For RNA Analysis:** Fresh tissue samples will be immediately immersed in RNALater solution (Thermo Fisher Scientific) and stored at -80°C within 2 hours of collection. This method preserves RNA integrity for up to 6 months and eliminates degradation associated with FFPE processing.
2. **For Histological Analysis:** Adjacent tissue sections will be fixed in 10% neutral buffered formalin for 24 hours followed by standard paraffin embedding for histological and immunohistochemical analyses.
3. **For Protein Analysis:** Fresh frozen samples will be snap-frozen in liquid nitrogen and stored at -80°C for protein extraction and Western blot analysis.

**RNA Quality Control:** RNA integrity will be assessed using Agilent 2100 Bioanalyzer with RNA Integrity Number (RIN) ≥7.0 required for inclusion in qPCR analysis.

### Quantitative reverse transcription polymerase chain reaction analysis

Immediately after harvesting vaginal tissue samples, a part of the samples will be immediately frozen in liquid nitrogen and kept at −80 °C until the RNA is extracted. The frozen tissue samples will be subjected to total RNA isolation using a commercial TRIzol reagent (Invitrogen, USA) and reverse transcribed using SensiFAST SYBR Lo-ROX Mix, 100 RXNS (BIOLINE) in accordance with the manufacturer’s instructions. RT-qPCR will be performed using an Applied Biosystems 7500 Fast System, Singapore. Subsequently, quantitative reverse transcription polymerase chain reaction (RT-qPCR) analysis will be conducted by using targeted primers for collagen type I, collagen type III, and MMP-1, in addition to a housekeeping gene (GAPDH) serving as an internal control. The relative expression levels of the target genes will be determined using the 2 ^−ΔΔCt^ method and standardized to the control group. The real-time PCR primer sequences are detailed in Table 1. The PCR primers will be constructed using the rat reference mRNA sequence from the National Center for Biotechnology Information (NCBI) database. Their specificity will be verified using the BLAST software from the NCBI website ( https://blast.ncbi.nlm.nih.gov/).

**Table 1.** Sequences of primers that will be utilized in the analysis of real-time PCR.

Gene	Primer sequence	Product size (bp)	Annealing Temp (°C)
COL1A1	**F:** ATC AGC CCA AAC CCC AAG GAG A **R:** CGC AGG AAG GTC AGC TGG ATA G	**156**	**60**
COL3A1	**F:** TGA TGG GAT CCA ATG AGG GAG A **R:** GAG TCT CAT GGC CTT GCG TGT TT	**184**	**60**
MMP1	**F:** TTG TTG CTG CCC ATG AGC TT **R:** ACT TTG TCG CCA ATT CCA GG	**142**	**60**
GAPDH	**F:** AACGACCCCTTCATTGAC **R:** TCCACGACATACTCAGCAC	**191**	**58**
B-Actin	**F:** CACGATGGAGGGGCCGGACTCATC **R:** TAAAGACCTCTATGCCAACACAGT	**240**	**60**

F: forward, R: reverse.

### Immunohistochemical analysis

The immunohistochemical examination will involve treating the deparaffinized and rehydrated paraffin-embedded vaginal tissue sections with a citrate-based buffer for antigen retrieval. Subsequently, the sections will be exposed to primary antibodies at a 1:200 dilution, specifically targeting collagen I alpha 2 Rabbit pAb (ABclonal, A5786), collagen III alpha 1 Rabbit pAb (ABclonal, A3795), and MMP-1 Rabbit pAb (Bioss, bs-0463R). Thereafter, they will be incubated with a 1:200 dilution of anti-rabbit secondary antibody (Servicebio) coupled with a chromogenic or fluorescent reporter at room temperature (RT). Microscopic assessment will be performed using a Leica microscope at 400× magnification by assessing positive brown staining of the stromal lamina propria of the vaginal mucosa in representative areas. For each sample, 5 different fields of view will be observed. In each unit area of the field of view (40× objective), photomicrographs will be taken using a DP-20 digital camera microscope with the aid of the DP2-BSW program. The proportion of the colored area will be measured with the ImageJ program. The results will be quantified as the proportion of the area with positive staining in relation to the overall area of the tissue.

### Sirius red staining

The vaginal tissue will be fixed in a 10% formalin solution for 24 hours, then embedded in paraffin and sliced into sections that are 5 micrometers thick. The vaginal tissue sections will be stained using standard procedures for Sirius Red staining. The slides stained with Sirius Red (0.1 g/100 mL saturated picric acid solution) (Sigma-Aldrich, USA) will be analyzed using a polarizing light microscope, which enables the observation of collagen fibers with improved birefringence characteristics. Collagen fibers that are arranged in an orderly manner will appear luminous and exhibit polarization, whereas fibers that are disorganized or broken will show a less intense or non-polarized appearance. The Sirius Red-stained sections will be evaluated by quantitative analysis utilizing ImageJ. The total area encompassed by collagen fibers, along with the proportion of structured (polarized) to unstructured (non-polarized) collagen fibers, will be calculated for every treatment group.

RT-qPCR, immunohistochemical, and Sirius Red staining data will be used to provide a comprehensive assessment of the effects of hPRF-L treatment on ECM composition and remodeling in the rat model of POP.

### Statistical analysis

The data obtained from RT-qPCR and immunohistochemical analyses will be presented as mean ± standard deviation. Statistical analyses will be conducted using computer programme. The data will undergo normality testing using the Shapiro–Wilk test, and the assumption of variance homogeneity will be confirmed using Levene’s test. If the assumptions are not met, suitable non-parametric tests, including the Kruskal–Wallis test, will be used. Differences among the control, sham, and hPRF-L treatment groups will be compared using one-way analysis of variance (ANOVA). If ANOVA indicates a statistically significant difference, subsequent post-hoc tests, including Tukey’s honestly significant difference or Dunnett’s test, will be used to determine the specific differences between groups. Statistical significance will be considered at p < 0.05.

## Discussion

The primary aim of this study is to examine the effect of hPRF-L injection on collagen type I, collagen type III, and MMP-1 levels in the vaginal mucosa of a rat model with POP. Our hypothesis is that hPRF-L application will positively affect the expression of important ECM components in a rat model of POP. We hypothesize that the hPRF-L treatment group will show a significant increase in collagen type I and III expression compared with the control and sham groups.

The rationale for this idea is based on the established functions of collagen type I and III in maintaining the structural integrity and biomechanical characteristics of pelvic floor tissues. Collagen type I is known to be exceptionally resistant to stretching and offers strength and stability to connective tissues, whereas collagen type III plays a role in providing elasticity and flexibility. Collectively, they preserve the ideal equilibrium required for efficient pelvic support. In POP cases, the ratio of these two collagen subtypes is often imbalanced. This imbalance involves a proportional reduction in collagen type I and an associated increase in collagen type III. The decline in collagen type I leads to a loss in tensile strength, rendering tissues less capable of enduring mechanical stress. Concurrently, an increase in collagen type III could affect the flexibility of tissues, making them more prone to stretching and deformation. This imbalance eventually damages the integrity of the pelvic support structures, leading to the onset and advancement of POP. ^18^^–^^20^

Moreover, we hypothesize that hPRF-L application will result in a simultaneous reduction of MMP-1 in the vaginal wall of a rat model with POP. MMP-1, also known as interstitial collagenase, is a crucial enzyme that breaks down collagen fibers in the ECM. The rationale for this idea is derived from the established function of MMP-1 in POP development. In POP, the excessive breakdown and remodeling of collagen-rich pelvic floor tissues is often attributed to increased MMP-1 activity. The imbalance between collagen production and degradation ultimately leads to the weakening and reduced structural integrity of vaginal and pelvic support systems. ^11^^,^^21^ Assuming that hPRF-L treatment will reduce MMP-1 production, we anticipate that this regenerative method will help control excessive ECM degradation. Reducing the activity of MMP-1 is expected to preserve the structural and functional integrity of the vaginal mucosa, which is important for providing support to the pelvic organs and preventing POP development or progression.

Recent clinical studies have demonstrated promising outcomes for PRP therapy in pelvic floor disorders. A systematic review by Prodromidou et al. (2022) found that in patients with POP, an increase in collagen concentration after PRP application was observed while the use of PRP resulted in improvement of stress urinary incontinence symptoms. ^22^ A recent systematic review reported high subjective improvement following PRP treatment in both stress urinary incontinence and female sexual dysfunction, with PRP appearing to have no significant adverse effects. ^23^ Clinical evidence shows favorable results with low recurrence rates in POP patients who received platelet-rich plasma compared to those who did not, with one-year follow-up studies finding no recurrences after patients received PRP at the surgical site. ^24^

The mechanism of PRP efficacy appears related to high concentrations of growth factors including PDGF, TGF-β, and VEGF, which stimulate collagen synthesis and angiogenesis. Studies have demonstrated that PRP increased the expression of type I collagen and MMP-1 in human dermal fibroblasts, indicating its role in extracellular matrix remodeling. However, while PRP has been proposed to enhance surgical outcomes of pelvic organ prolapse by native tissue repair with promising success rates, optimal preparation methods, injection techniques, and treatment intervals remain undefined. ^25^

Our study utilizing hPRF-L represents an advancement over traditional PRP, as the lysate preparation releases growth factors more consistently and provides sustained bioavailability. The rat model will enable mechanistic investigation of collagen remodeling pathways that cannot be ethically studied in humans, providing crucial translational data for optimizing clinical protocols.

The rationale behind this hypothesis **is that** the wide variety of growth factors, cytokines, and other biologically active molecules found in hPRF-L **will promote** collagen production, **inhibit** MMP activity, and **facilitate** the overall regeneration and restructuring of pelvic floor tissues. These biologically active compounds **can increase** cell growth and differentiation, thereby **aiding** the restoration of impaired or weakened tissues. Thus, hPRF-L treatment **offers** a potential therapeutic approach for addressing the underlying structural deficiencies that contribute to POP development and progression by restoring the ideal balance of these crucial ECM components and regulators. ^26^^,^^27^ The goal of this technique **is to strengthen** pelvic floor tissues, **improve** their biomechanical properties, and **reduce** the likelihood of future recurrence or progression of POP.

Compared with platelet-rich plasma (PRP), the hPRF-L used in this study is a more advanced and possibly more efficient method for regenerative therapy in POP, surpassing the first-generation platelet concentrate technology. Unlike PRP, which requires the use of anticoagulants and external thrombin to activate platelets, hPRF-L is produced using a more natural method that uses the patient’s own blood coagulation. The intrinsic coagulation process enables the entrapment of a broader range of growth factors, cytokines, and other biologically active substances within the fibrin structure. Subsequently, these substances are gradually released over a period of time to facilitate tissue regeneration and restructuring. Furthermore, the absence of anticoagulants and the limited use of centrifugation processes in the hPRF-L preparation protocol aid in maintaining the integrity and functionality of the platelets and their associated biomolecules. ^14^^,^^15^^,^^28^ This can improve the therapeutic effectiveness of hPRF-L compared with PRP. Moreover, the fibrin-based composition of hPRF-L offers a more enduring and consistent release of bioactive substances. This characteristic can be beneficial in managing the persistent and advancing nature of POP.

The findings of this study on the effects of human platelet-rich fibrin lysate (hPRF-L) on extracellular matrix components in a rat model of pelvic organ prolapse (POP) may have broader implications, but several considerations must be taken into account when generalizing to other species or conditions:

1. Species differences: While rats are commonly used in POP research, their quadrupedal nature and differences in pelvic floor anatomy and biomechanics limit direct translation to bipedal humans. However, the fundamental processes of extracellular matrix remodeling and the response to growth factors are likely to be conserved across mammals.
2. Relevance to human biology: The molecular pathways involved in collagen synthesis and matrix metalloproteinase regulation are highly conserved in mammals. Therefore, the effects of hPRF-L on these pathways in rats may provide valuable insights into potential human applications. However, the complexity of human POP, influenced by factors such as genetic predisposition, lifestyle, and hormonal status, may not be fully captured in this model.
3. Experimental conditions: The controlled environment of our study, while necessary for scientific rigor, may not fully replicate the variability seen in clinical settings. Factors such as age, comorbidities, and varying degrees of POP severity could influence treatment efficacy in humans.
4. Potential for translation: Despite these limitations, our study provides a crucial step in evaluating the potential of hPRF-L as a regenerative therapy for POP. The molecular and histological outcomes we assess are directly relevant to the pathophysiology of human POP and could inform future preclinical and clinical studies.
5. Broader applications: The effects of hPRF-L on collagen synthesis and MMP regulation may have implications beyond POP, potentially extending to other conditions involving connective tissue disorders or wound healing.

The findings of this study **are expected to provide** valuable insights into the potential mechanisms by which hPRF-L **may exert** its beneficial effects on the vaginal mucosa in the context of POP. By demonstrating the positive effect of hPRF-L on the expression of key ECM components and MMP regulation, this research **lays** the groundwork for further investigations into the therapeutic applications of this regenerative approach for the management of POP.

### Strength and Limitation of this study

> The study will employ a thoroughly characterized rat model of pelvic organ prolapse, which will offer a controlled and replicable system for assessing the impacts of hPRF-L therapy.
> The study will utilize a multi-modal approach, including histological (Sirius Red staining), molecular (RT-qPCR), and immunohistochemical techniques, to thoroughly evaluate the impact of hPRF-L on collagen deposition, organization, and the expression of important extracellular matrix components.
> The study will aim to determine if there are dose-dependent effects and provide insights into the most effective dosing regimens for future clinical applications.
> Translation of animal models will be considered.
> The study will examine the impact of hPRF-L therapy throughout a relatively brief duration.
> The study will not include biomechanical testing.

#### Ethics and consent

This study protocol was reviewed and approved by the Ethics Commission of the Faculty of Medicine, Public Health and Nursing, Gadjah Mada University (FKKMK UGM) on [18 January 2023] (ethics commission approval letter with reference number: KE/FK/0092/EC/2023). This approval covers both the animal study and the use of human blood products.

Human Participants: The study adheres to the principles of the Declaration of Helsinki. All participants providing blood samples for hPRF-L preparation will undergo an informed consent process. Written informed consent will be obtained from each participant after a thorough explanation of the study’s purpose, procedures, risks, and benefits. Participants will be given ample time to consider their involvement and ask questions. All participant information will be kept confidential and de-identified.

Animal Welfare: All procedures involving animals will be conducted in strict compliance with the guidelines set forth in the Guide for the Care and Use of Laboratory Animals published by the National Institutes of Health (NIH). Every effort will be made to minimize animal suffering and distress throughout the study. These efforts include:

1. Use of appropriate anesthesia (ketamine/xylazine) for all surgical procedures, with continuous monitoring of anesthetic depth.
2. Regular monitoring of animals for signs of distress, with implementation of humane endpoints if necessary.
3. Housing animals in a temperature-controlled environment with a 12-hour light/dark cycle and free access to food and water.
4. Limiting the duration of the simulated vaginal delivery procedure to 4 hours to minimize stress.
5. Gentle handling: All procedures, including hPRF-L injections, were performed by trained personnel to minimize stress and discomfort.
6. Euthanasia will be performed using methods approved by the American Veterinary Medical Association (AVMA) Guidelines for the Euthanasia of Animals.

The number of animals used in this study has been carefully calculated to achieve statistical significance while using the minimum number of animals possible. All personnel involved in animal handling and procedures have received appropriate training in laboratory animal science and welfare.

The results of this study will be disseminated through various channels to enhance scientific knowledge in the area of pelvic organ prolapse and regenerative medicine. Upon completion of the study, the research team intends to publish the findings in a peer-reviewed scientific journal. This will enable professionals in the field to critically assess the findings and ensure the study’s scientific rigor and validity. Additionally, the research team will disseminate the study findings by presenting them at relevant national and international conferences and symposia. These platforms will facilitate the dissemination of knowledge acquired from this study to the wider scientific community and foster meaningful discussions and partnerships with other researchers in related fields.

## Data Availability

No data are associated with this article. OSF Repository: ARRIVE checklist for “Effect of Human Platelet-Rich Fibrin Lysate on Collagen Type I, Collagen Type III, and Matrix Metalloproteinase 1 in Rat Models with Pelvic Organ Prolapse”. https://doi.org/10.17605/OSF.IO/QE6KU. ^29^ Data are available under the terms of the Creative Commons Attribution 4.0 International license (CC-BY 4.0).
