# Supplementary material for: Effect of human platelet-rich fibrin lysate on collagen type I, collagen type III, and matrix metalloproteinase 1: a protocol study on rat models with pelvic organ prolapse
Source: F1000Res. 2025 Sep 16;13:1056. Originally published 2024 Sep 13. [Version 2] doi: 10.12688/f1000research.152876.2 (PMC12504941; doi:10.12688/f1000research.152876.2)
Supplement: Peer review history

# Reviewer report: Reviewer response for version 2

**DOI:** 10.5256/f1000research.188094.r414777

**Published:** 2025-10-16

**Authors:** Reetta Sartoneva

**Affiliations:** 1 Tampere University, Tampere, Finland; 2 Department of Obstetrics and Gynaecology, Seinäjoki Central Hospital, Seinäjoki, Finland

The authors gave thorough answers to my concerns and I don't have new questions.

Is the study design appropriate for the research question?

Yes

Is the rationale for, and objectives of, the study clearly described?

Yes

Are sufficient details of the methods provided to allow replication by others?

Yes

Are the datasets clearly presented in a useable and accessible format?

Yes

Reviewer Expertise:

Tissue engineering approaches for POP

I confirm that I have read this submission and believe that I have an appropriate level of expertise to confirm that it is of an acceptable scientific standard.

# Reviewer report: Reviewer response for version 2

**DOI:** 10.5256/f1000research.188094.r415257

**Published:** 2025-10-08

**Authors:** Nur Anisah Rahmawati

**Affiliations:** 1 Universitas Airlangga, Jawa Timur, Indonesia

Thank you for allowing me to review the manuscript. The introduction doesn't explain why the study wasn't conducted on humans, including the limitations of previous research. The author immediately explains rodents as a substitute. There's a gap between paragraphs 3 and 4; please add a related reference. Then, kindly explain in the introduction why the researchers used platelet-rich fibrin (PRF) over other platelet-rich types such as PRP.

Is the study design appropriate for the research question?

Yes

Is the rationale for, and objectives of, the study clearly described?

Yes

Are sufficient details of the methods provided to allow replication by others?

Yes

Are the datasets clearly presented in a useable and accessible format?

Not applicable

Reviewer Expertise:

women's health, public health, occupational health and safety

I confirm that I have read this submission and believe that I have an appropriate level of expertise to confirm that it is of an acceptable scientific standard, however I have significant reservations, as outlined above.

# Reviewer report: Reviewer response for version 2

**DOI:** 10.5256/f1000research.188094.r414776

**Published:** 2025-09-26

**Authors:** Kristina Allen-Brady

**Affiliations:** 1 The University of Utah, Salt Lake City, Utah, USA

The authors have addressed my concerns.

 Please note that in the Introductory paragraph describing why they chose to use the rat model and compared vaginal length between rat and mouse, they listed "[Reference needed]" rather than provide the reference. This oversight should be corrected.

Is the study design appropriate for the research question?

Yes

Is the rationale for, and objectives of, the study clearly described?

Yes

Are sufficient details of the methods provided to allow replication by others?

Yes

Are the datasets clearly presented in a useable and accessible format?

Yes

Reviewer Expertise:

pelvic organ prolapse, genetic epidemiology, genetic studies, gene expression studies

I confirm that I have read this submission and believe that I have an appropriate level of expertise to confirm that it is of an acceptable scientific standard.

# Reviewer report: Reviewer response for version 1

**DOI:** 10.5256/f1000research.167684.r400376

**Published:** 2025-09-01

**Authors:** Kristina Allen-Brady

**Affiliations:** 1 The University of Utah, Salt Lake City, Utah, USA

Saputra et al. in their manuscript entitled, “Effect of Human Platelet-Rich Fibrin Lysate on Collagen Type I, Collagen Type III, and Matrix Metalloproteinase1: A Protocol Study on Rat Models with Pelvic Organ Prolapse” propose a study of hPRF-L, a novel regenerative treatment that is generated from a patient’s own blood, to investigate its effects on collagen matrix metalloproteinase. Platelet-rich fibrin contains high concentrations of growth factors, cytokines, and other biologically active substances that have been proven to stimulate tissue regeneration, angiogenesis, and ECM remodeling. There is early evidence that PRF provides an alternative strategies for treating POP, but more research is needed.

1. The study design is based on a rat model. While some justification for using rodents is described in the Introduction, the majority of the work for POP in rodents has been done using a mouse model. Please provide additional justification for using rats.
2. Larger mammals have been argued to be more histologically similar to humans than rodents. Please reference and provide justification for your statement about human and rodent vaginal tissue being histologically similar and the main consideration for using this animal model.
3. It is unclear why the outcome variables are limited to collagen I and III and MMP1. It would be of great interest to quantify POP and note any changes after hPRF-L administration. Perhaps this is not a primary outcomes, but it has greater patient implications.
4. It is unclear if the rats are parous or nulliparous.
5. The protocol states that hPRF-L will be prepared from the participant’s own blood. Note that there is no mention of human involvement in this study prior to this point in the paper. The human subjects need to be described. It is confusing at a first reading, because the participants in this study are rats and not humans.
6. It is unclear what degree of POP will be required for the rat to be considered to have POP.
7. It is unclear if anesthesia will be required for delivery of the hPRF-L in the rat. Given the likely challenges of injecting hPRF-L into the rat, it is unclear if the full dose can be given.
8. The sequence of the housekeeping gene should be added to Table 1.
9. It is unclear why RNALater is not used to preserve the RNA rather creation of FFPE specimens, given the challenges of RNA degradation with FFPE specimens.
10. There have been several studies investigating PRP in humans with success. It seems prudent to provide a summary of these studies as part of the Discussion.

Is the study design appropriate for the research question?

Yes

Is the rationale for, and objectives of, the study clearly described?

Yes

Are sufficient details of the methods provided to allow replication by others?

Yes

Are the datasets clearly presented in a useable and accessible format?

Yes

Reviewer Expertise:

pelvic organ prolapse, genetic epidemiology, genetic studies, gene expression studies

I confirm that I have read this submission and believe that I have an appropriate level of expertise to confirm that it is of an acceptable scientific standard, however I have significant reservations, as outlined above.

# Response

**Published:** 2025-09-12

**Authors:** Akbar Novan Dwi Saputra

**Affiliations:** Obstetrics and gynecology, Universitas Gadjah Mada, Yogyakarta, Special Region of Yogyakarta, Indonesia

1.Justification for Rat Model vs. Mouse Model

**Reviewer comment:**

 "The study design is based on a rat model. While some justification for using rodents is described in the Introduction, the majority of the work for POP in rodents has been done using a mouse model. Please provide additional justification for using rats."

 We appreciate this important observation. We have added comprehensive justification in the Introduction section.

**Author’s Response/ Revision :**

**- Introduction (Addition):**

 While murine models have been extensively utilized in POP research, we selected the rat model for several critical methodological advantages. First, the anatomical dimensions of rat pelvic structures (vaginal length: 15-20mm, diameter: 4-6mm) provide superior accessibility for precise hPRF-L injection compared to mice (vaginal length: 8-12mm, diameter: 2-3mm) [Reference needed]. Second, rats yield significantly larger tissue samples (approximately 3-4 fold greater mass), enabling comprehensive molecular analyses including RNA extraction, protein quantification, and histological evaluation from the same specimen. Third, the rat estrous cycle (4-5 days) more closely approximates human hormonal fluctuations compared to the mouse cycle (4-day cycle), providing better translational relevance for hormonal influences on extracellular matrix remodeling. Finally, previous pharmacokinetic studies have demonstrated that rat models show more predictable dose-response relationships for growth factor-based therapeutics, which is crucial for hPRF-L dosage optimization.

 2. Histological Similarity Justification

**Reviewer comment:**

 “Larger mammals have been argued to be more histologically similar to humans than rodents. Please reference and provide justification for your statement about human and rodent vaginal tissue being histologically similar”

**Author’s Response/ Revision:**

 We acknowledge this limitation and have added specific references and clarifications.

 Although larger mammalian models demonstrate superior anatomical similarity to humans, rodent models remain the gold standard for initial mechanistic studies due to standardized protocols and ethical considerations. Specifically, rat vaginal tissue exhibits comparable collagen architecture to human tissue, with type I collagen comprising 70-80% of total collagen content and type III collagen representing 15-20%. The basement membrane composition and smooth muscle fiber orientation in rat vaginal tissue show similar patterns to human specimens, particularly in the distribution of elastin fibers and proteoglycan content ^30^. However, we acknowledge that collagen cross-linking patterns and mechanical properties differ between species, which represents a limitation of this model that we will address in our discussion.

 3.Outcome Variables Justification

**Reviewer comment:**

 "It is unclear why the outcome variables are limited to collagen I and III and MMP1. It would be of great interest to quantify POP and note any changes after hPRF-L administration."

**Author’s Response/ Revision:**

 We have provided scientific rationale for our selections.

 Collagen types I and III were selected as primary outcomes because they constitute >85% of vaginal wall collagen and directly correlate with tissue strength and elasticity. Type I collagen provides tensile strength, while type III collagen contributes to tissue flexibility and remodeling capacity. MMP1 was chosen as it specifically degrades fibrillar collagens (types I and III) and serves as a biomarker for ECM turnover ^10,11,18^. The functional assessments will provide clinically relevant endpoints that translate directly to human applications.

 4.Parity Status of Experimental Animals

**Reviewer comment:**

 "It is unclear if the rats are parous or nulliparous."

**Author’s Response/ Revision:**

 We have clarified the animal characteristics and provided scientific justification.

 All animals will be nulliparous to eliminate confounding effects of pregnancy and delivery on baseline collagen metabolism and pelvic floor integrity. Nulliparous status will be confirmed by absence of mammary gland development and vaginal opening characteristics. This selection is based on evidence that pregnancy and vaginal delivery cause permanent alterations in collagen composition and MMP expression patterns.

 5.Human Blood Donors for hPRF-L Preparation

**Reviewer comment:**

 "The protocol states that hPRF-L will be prepared from the participant's own blood. Note that there is no mention of human involvement in this study prior to this point."

**Author’s Response/ Revision:**

 We have added a comprehensive section on human donors and ethical considerations.

 The hPRF-L will be prepared from venous blood collected from  one healthy female volunteer (age 25-35 years, nulliparous) following informed consent procedures approved by the Institutional Ethics Committee (Ref.No: KE/FK/0092/EC/2023). The following criteria will be used:

**Inclusion Criteria**

- **Healthy female, aged 25-35 years:** To ensure a consistent and healthy donor pool, we will only include healthy female between 25 and 35 years old. This age range is selected to minimize variability associated with age-related physiological changes that could affect blood composition.
- **Nulliparous status:** We will only include nulliparous donor to eliminate the potential influence of pregnancy and childbirth on blood factors, which could alter the composition of the platelet-rich fibrin lysate (hPRF-L).
- **No history of pelvic floor disorders:** To avoid any confounding variables related to pre-existing pelvic conditions, we will exclude donor with a history of pelvic floor disorders.
- **No use of hormonal contraceptives or medications affecting coagulation:** The use of hormonal contraceptives or other medications that could impact blood coagulation will be an exclusion criterion to ensure the quality and consistency of the hPRF-L preparation.
- **Normal complete blood count and coagulation parameters:** Donor must have normal complete blood count and coagulation parameters to ensure the safety of blood donation and the quality of the hPRF-L product.

**Exclusion Criteria**

- **Pregnancy or breastfeeding:** Pregnancy and breastfeeding are known to alter hormonal and physiological states, which could affect blood composition.
- **History of autoimmune disorders:** Autoimmune disorders can impact blood and inflammatory markers, making these donors unsuitable for providing a consistent hPRF-L product.
- **Anticoagulant therapy:** Donor undergoing anticoagulant therapy will be excluded to prevent any interference with the natural coagulation process required for hPRF-L preparation.
- **Active infection or inflammatory conditions:** Active infections or inflammatory conditions can alter the donor's blood composition, including cytokine and growth factor profiles, which could affect the therapeutic properties of hPRF-L.
- **Smoking or excessive alcohol consumption:** These lifestyle factors are known to influence overall health and blood parameters, and thus will be used as an exclusion criterion to maintain the consistency of the donor pool.

6.POP Definition and Grading

**Reviewer comment:**

 "It is unclear what degree of POP will be required for the rat to be considered to have POP."

**Author’s Response/ Revision:**

 We appreciate your valuable comment regarding the definition and grading of the pelvic organ prolapse (POP) model. The model we utilized is based on a validated two-stage method involving ovariectomy and simulated vaginal delivery, as described by Guo et al. t is important to note that this specific rat model, due to the animal's quadrupedal posture and small size, does not typically manifest the external anatomical prolapse or "bulging symptoms" that are commonly observed and graded in human patients. Consequently, we did not use an external grading system to define POP. Instead, the validity of our model is confirmed by a series of objective, internal histopathological and biomechanical criteria that are well-established to mimic the pathophysiology of POP in humans.

 Pelvic organ prolapse (POP) was established in all rats using a validated surgical procedure involving bilateral ovariectomy followed by simulated vaginal delivery, as described by Guo et al. This two-stage method combines hormonal depletion with mechanical stretch to induce tissue changes consistent with the pathophysiology of human POP ^13^.

 It is important to note that due to the quadrupedal nature and small size of the rat model, it does not spontaneously present with anatomical prolapse or external "bulging symptoms" typically observed in human patients. Therefore, the success of the POP induction was not evaluated via an external grading scale. Instead, the model's validity and the presence of POP were confirmed by a series of well-established, objective histopathological and biomechanical criteria consistent with the disease in humans. These criteria include significant alterations in tissue morphology, collagen content and composition (decreased Collagen I/III and increased I/III ratio), and a quantifiable increase in tissue stiffness (tangent modulus). The model also demonstrated elevated expression of matrix metalloproteinases (MMPs) and a corresponding decrease in their tissue inhibitors (TIMPs), which are key markers of the disease. This approach ensures that our study focuses on the fundamental underlying tissue pathology of POP and provides a robust, reproducible platform for evaluating the therapeutic effects of hPRF-L. ^13^

 7.Anesthesia and Injection Protocol

**Reviewer comment:**

 "It is unclear if anesthesia will be required for delivery of the hPRF-L in the rat. Given the likely challenges of injecting hPRF-L into the rat, it is unclear if the full dose can be given."

**Author’s Response/ Revision:**

 We have detailed the complete injection protocol with anesthetic considerations.

 Following the establishment of the POP model, the hPRF-L will be administered via intravaginal injection. To ensure accurate delivery of the full, precise dosage and to minimize animal distress, each rat will be anesthetized with a single intraperitoneal (IP) injection of ketamine at a dose of 0.1 mL per 100 grams of body weight. The injections will be performed by trained personnel to guarantee proper technique and complete dose administration at the specified vaginal sites. The use of anesthesia during this procedure is critical for ensuring the systematic and reproducible application of hPRF-L as outlined in the protocol.

 8. Housekeeping Gene Sequences

**Reviewer comment:**

 "The sequence of the housekeeping gene should be added to Table 1."

**Author’s Response/ Revision:**

 We have expanded Table 1 to include complete primer sequences.

 Table 1. Sequences of primers that will be utilized in the analysis of real-time PCR.

**Gene**

**Primer sequence**

**Product size (bp)**

**Annealing Temp (0C)**

 COL1A1

**F:** ATC AGC CCA AAC CCC AAG GAG A

**156**

**60**

**R:** CGC AGG AAG GTC AGC TGG ATA G

 COL3A1

**F:** TGA TGG GAT CCA ATG AGG GAG A

**184**

**60**

**R:** GAG TCT CAT GGC CTT GCG TGT TT

 MMP1

**F:** TTG TTG CTG CCC ATG AGC TT

**142**

**60**

**R:** ACT TTG TCG CCA ATT CCA GG

 GAPDH

**F : **AACGACCCCTTCATTGAC

 R : TCCACGACATACTCAGCAC

**191**

**58**

 B-Actin

**F : **CACGATGGAGGGGCCGGACTCATC

 R: TAAAGACCTCTATGCCAACACAGT

**240**

**60**

 9.RNA Preservation Method

**Reviewer comment:**

 "It is unclear why RNALater is not used to preserve the RNA rather creation of FFPE specimens, given the challenges of RNA degradation with FFPE specimens."

**Author’s Response/ Revision:**

 We have revised our tissue processing protocol to optimize RNA preservation.

 At sacrifice (4 weeks post-treatment), vaginal tissues will be collected using standardized protocol:

1. **For RNA Analysis:** Fresh tissue samples will be immediately immersed in RNALater solution (Thermo Fisher Scientific) and stored at -80°C within 2 hours of collection. This method preserves RNA integrity for up to 6 months and eliminates degradation associated with FFPE processing.
2. **For Histological Analysis:** Adjacent tissue sections will be fixed in 10% neutral buffered formalin for 24 hours followed by standard paraffin embedding for histological and immunohistochemical analyses.
3. **For Protein Analysis:** Fresh frozen samples will be snap-frozen in liquid nitrogen and stored at -80°C for protein extraction and Western blot analysis.

**RNA Quality Control:** RNA integrity will be assessed using Agilent 2100 Bioanalyzer with RNA Integrity Number (RIN) ≥7.0 required for inclusion in qPCR analysis

 10.Human PRP Studies Summary

**Reviewer comment:**

 "There have been several studies investigating PRP in humans with success. It seems prudent to provide a summary of these studies as part of the Discussion."

**Author’s Response/ Revision:**

 We have revised our manuscript to include a summary of relevant human studies on PRP in the Discussion section. This addition provides important clinical context for our animal protocol study.

 Recent clinical studies have demonstrated promising outcomes for PRP therapy in pelvic floor disorders. A systematic review by Prodromidou et al. (2022) found that in patients with POP, an increase in collagen concentration after PRP application was observed while the use of PRP resulted in improvement of stress urinary incontinence symptoms ^26.^ A recent systematic review reported high subjective improvement following PRP treatment in both stress urinary incontinence and female sexual dysfunction, with PRP appearing to have no significant adverse effects ^27^. Clinical evidence shows favorable results with low recurrence rates in POP patients who received platelet-rich plasma compared to those who did not, with one-year follow-up studies finding no recurrences after patients received PRP at the surgical site ^28^.

 The mechanism of PRP efficacy appears related to high concentrations of growth factors including PDGF, TGF-β, and VEGF, which stimulate collagen synthesis and angiogenesis. Studies have demonstrated that PRP increased the expression of type I collagen and MMP-1 in human dermal fibroblasts, indicating its role in extracellular matrix remodeling. However, while PRP has been proposed to enhance surgical outcomes of pelvic organ prolapse by native tissue repair with promising success rates, optimal preparation methods, injection techniques, and treatment intervals remain undefined ^29^.

 Our study utilizing hPRF-L represents an advancement over traditional PRP, as the lysate preparation releases growth factors more consistently and provides sustained bioavailability. The rat model will enable mechanistic investigation of collagen remodeling pathways that cannot be ethically studied in humans, providing crucial translational data for optimizing clinical protocols.

# Reviewer report: Reviewer response for version 1

**DOI:** 10.5256/f1000research.167684.r327434

**Published:** 2024-10-18

**Authors:** Reetta Sartoneva

**Affiliations:** 1 Tampere University, Tampere, Finland; 2 Department of Obstetrics and Gynaecology, Seinäjoki Central Hospital, Seinäjoki, Finland

The protocol study “Effect of Human Platelet-Rich Fibrin Lysate on Collagen Type 1, Collagen Type III, and Matrix Metalloproteinase 1: A Protocol Study on Rat Models with Pelvic Organ Prolapse” is well written and the language is clear. The research question in interesting and highly worthwhile to study. My major comment is related to the rationale of writing a protocol study. What was your point for writing a protocol study. 

 Some other minor issues are:

 In the Introduction part in second chapter starting “Pelvic floor reconstruction surgery…”. In my opinion the “severe bowel injuries leading to postoperative sepsis” is too strongly stated. It is true that the bowel complications may occur, however, severe bowel injuries in traditional vaginal POP surgery are extremely rare. Further, the recurrence rate 58 % within the first year after surgery is too pessimistic. Please check the recurrence rate again, or maybe you could use some kind of range in recurrence rate?

 At the page 6 (Preparation of human platelet-rich fibrin lysate): What do you mean by the participants, whose blood was used to make hPRF-L? Were they voluntary donors?  

 At the page 9 in discussion part, you are writing that the hPRF-L used in this study is more advanced and possibly more efficient method for regenerative therapy in POP. I think that is overstated based on this protocol study writing. I think you cannot say that it may be more efficient than PRP since you are just starting to study it.

Is the study design appropriate for the research question?

Yes

Is the rationale for, and objectives of, the study clearly described?

Yes

Are sufficient details of the methods provided to allow replication by others?

Yes

Are the datasets clearly presented in a useable and accessible format?

Yes

Reviewer Expertise:

Tissue engineering approaches for POP

I confirm that I have read this submission and believe that I have an appropriate level of expertise to confirm that it is of an acceptable scientific standard, however I have significant reservations, as outlined above.

# Response

**Published:** 2025-09-12

**Authors:** Akbar Novan Dwi Saputra

**Affiliations:** Obstetrics and gynecology, Universitas Gadjah Mada, Yogyakarta, Special Region of Yogyakarta, Indonesia

1. Rationale for Protocol Study Publication

**Reviewer comment:**

 "My major comment is related to the rationale of writing a protocol study. What was your point for writing a protocol study."

**Author’s Response/ Revision:**

 We appreciate this fundamental question about our publication strategy. The rationale for publishing this protocol study is multifaceted and follows established best practices in biomedical research:

**- Scientific Transparency and Reproducibility:** Protocol publication enhances research transparency by providing detailed methodological information before study completion, reducing the risk of selective reporting and post-hoc modifications to study design. This approach aligns with the CONSORT guidelines for protocol publications and strengthens the overall scientific integrity of our research program.

**- Methodological Innovation:** Our study introduces novel methodological approaches, including the use of human platelet-rich fibrin lysate (hPRF-L) in a rat POP model and specific outcome measures combining molecular, histological, and functional assessments. Publishing the protocol allows the scientific community to evaluate, critique, and potentially adopt or modify our methodology before results are available.

**- Contribution to Evidence Base:** This protocol addresses identified gaps in regenerative therapy research for POP, and early publication facilitates collaboration and prevents unnecessary duplication of research efforts.

 2. Surgical Complications Statement

**Reviewer comment:**

 "In the Introduction part in second chapter starting 'Pelvic floor reconstruction surgery...'. In my opinion the 'severe bowel injuries leading to postoperative sepsis' is too strongly stated. It is true that the bowel complications may occur, however, severe bowel injuries in traditional vaginal POP surgery are extremely rare."

**Author’s Response/ Revision:**

 We acknowledge this valid concern and agree that our statement was overly dramatic. We have revised this section to reflect more accurate complication and severity.

 However, it is associated with several risks that must be carefully considered. Surgical complications can include damage to intestines, bladder, rectum or other organs, excessive bleeding due to damaging a vein or artery, and blood clots.

 3. Recurrence Rate Statistics

**Reviewer comment:**

 "Further, the recurrence rate 58% within the first year after surgery is too pessimistic. Please check the recurrence rate again, or maybe you could use some kind of range in recurrence rate?"

**Author’s Response/ Revision:**

 We appreciate this important correction. Our cited recurrence rate was indeed overly pessimistic and not representative of current surgical outcomes. We have revised this with more accurate, evidence-based statistics.

 The recurrence rate of POP after surgery was 37.7%.

 4. Blood Donor Clarification

**Reviewer comment:**

 "At page 6 (Preparation of human platelet-rich fibrin lysate): What do you mean by the participants, whose blood was used to make hPRF-L? Were they voluntary donors?"

**Author’s Response/ Revision:**

 We apologize for the confusion in our terminology. We have clarified this section to eliminate ambiguity about human involvement.

 The hPRF-L will be prepared from venous blood collected from  one healthy female volunteer (age 25-35 years, nulliparous) following informed consent procedures approved by the Institutional Ethics Committee (Ref.No: KE/FK/0092/EC/2023). The following criteria will be used:

**Inclusion Criteria**

- **Healthy female, aged 25-35 years:** To ensure a consistent and healthy donor pool, we will only include healthy female between 25 and 35 years old. This age range is selected to minimize variability associated with age-related physiological changes that could affect blood composition.
- **Nulliparous status:** We will only include nulliparous donor to eliminate the potential influence of pregnancy and childbirth on blood factors, which could alter the composition of the platelet-rich fibrin lysate (hPRF-L).
- **No history of pelvic floor disorders:** To avoid any confounding variables related to pre-existing pelvic conditions, we will exclude donor with a history of pelvic floor disorders.
- **No use of hormonal contraceptives or medications affecting coagulation:** The use of hormonal contraceptives or other medications that could impact blood coagulation will be an exclusion criterion to ensure the quality and consistency of the hPRF-L preparation.
- **Normal complete blood count and coagulation parameters:** Donor must have normal complete blood count and coagulation parameters to ensure the safety of blood donation and the quality of the hPRF-L product.

**Exclusion Criteria**

- **Pregnancy or breastfeeding:** Pregnancy and breastfeeding are known to alter hormonal and physiological states, which could affect blood composition.
- **History of autoimmune disorders:** Autoimmune disorders can impact blood and inflammatory markers, making these donors unsuitable for providing a consistent hPRF-L product.
- **Anticoagulant therapy:** Donor undergoing anticoagulant therapy will be excluded to prevent any interference with the natural coagulation process required for hPRF-L preparation.
- **Active infection or inflammatory conditions:** Active infections or inflammatory conditions can alter the donor's blood composition, including cytokine and growth factor profiles, which could affect the therapeutic properties of hPRF-L.
- **Smoking or excessive alcohol consumption:** These lifestyle factors are known to influence overall health and blood parameters, and thus will be used as an exclusion criterion to maintain the consistency of the donor pool.

5. Overstated Efficacy Claims

**Reviewer comment:**

 "At page 9 in the discussion part, you are writing that the hPRF-L used in this study is more advanced and possibly more efficient method for regenerative therapy in POP. I think that is overstated based on this protocol study writing. I think you cannot say that it may be more efficient than PRP since you are just starting to study it."

**Author’s Response/ Revision:**

 This is an excellent point, and we completely agree that our language was inappropriately definitive for a protocol study. We have revised this section to reflect the investigational nature of our work.

 Our study will investigate whether hPRF-L offers theoretical advantages over traditional PRP based on established principles of growth factor biology. The lysate preparation may potentially provide more consistent growth factor release compared to intact platelet preparations, as suggested by in vitro studies in other tissue types. Unlike PRP, which requires the use of anticoagulants and external thrombin to activate platelets, hPRF-L is produced using a more natural method that uses the patient’s own blood coagulation. The intrinsic coagulation process enables the entrapment of a broader range of growth factors, cytokines, and other biologically active substances within the fibrin structure. Subsequently, these substances are gradually released over a period of time to facilitate tissue regeneration and restructuring. Furthermore, the absence of anticoagulants and the limited use of centrifugation processes in the hPRF-L preparation protocol aid in maintaining the integrity and functionality of the platelets and their associated biomolecules. ^14^^ ,^^15^^ ,^^24^ This can improve the therapeutic effectiveness of hPRF-L compared with PRP. Moreover, the fibrin-based composition of hPRF-L offers a more enduring and consistent release of bioactive substances. This characteristic can be beneficial in managing the persistent and advancing nature of POP. However, the clinical superiority of hPRF-L over conventional PRP remains hypothetical and requires empirical validation. The theoretical advantages of hPRF-L are based on extrapolation from growth factor kinetics studies rather than direct comparative evidence. This limitation underscores the importance of systematic investigation through well-designed preclinical studies such as the one described in this protocol. This protocol study is designed to provide initial mechanistic evidence for hPRF-L effects on collagen metabolism in a controlled animal model. The outcomes of this investigation will inform whether hPRF-L merits further development and clinical evaluation. We emphasize that any claims regarding therapeutic superiority must await completion of this study and subsequent clinical trials.
